# Satisfaction with in vitro fertilization treatment: patients’ experiences and professionals’ perceptions

**DOI:** 10.1186/s40738-016-0019-4

**Published:** 2016-04-02

**Authors:** Limor Dina Gonen

**Affiliations:** Department of Economics and Business Administration, Ariel University, Ariel, Israel

**Keywords:** Reproductive technology, In vitro fertilization (IVF), Patient’s satisfaction, Evaluation, Quality of care, Survey, Willingness to pay (WTP), Treatment experience, Psychological factors

## Abstract

**Background:**

This paper investigates patients’ satisfaction with various aspects of fertility care and seeks to determine to what extent fertility specialists are able to assess patient satisfaction. Patients’ experiences with in-vitro fertilization (IVF) services and facilities have been compiled and examined in order to discover whether patients’ satisfaction is correlated to psychological factors and demographic, socio-economic, and health characteristics, and whether patients’ satisfaction has an influence on the willingness to pay (WTP) for IVF treatment.

**Methods:**

The study was carried out on 204 patients and 19 fertility professionals from 8 public IVF units in Israel.

**Results:**

The study found that, overall, infertile patients are satisfied with the care they received. Several demographic variables (age; education; income; number of fertility treatments) and psychological factors (‘Pessimism’ and ‘Activeness’), were found to be significantly correlated with patient satisfaction with IVF.

The results yielded a negative correlation between the WTP for IVF treatment and the satisfaction with access to care and physical conditions.

**Conclusions:**

Patient satisfaction is an important component in the evaluation of fertility treatments as well as other medical interventions. Insights into the quality of care as seen from the patients’ perspective may help healthcare staff better meet patients’ needs, wishes, and priorities.

## Background

The evaluation of patients’ experiences and needs in health care is a vital component in assessing quality of care [1–4], especially regarding fertility care [5–8]. Although medically assisted reproduction has been having appreciable success, an additional exploration of the issue of patient satisfaction is justified because: (i) one-third of the infertile couples ultimately do not deliver a child [9, 10]. Consequently, in addition to outcome indicators, process indicators, such as patients’ satisfaction, are very important. (ii) Infertility and the attendant medical interventions impose physical and emotional burdens on women and men alike [11–14], which in turn impact drop-out rates [15, 16]. The lack of attention to patients’ emotional and physical needs may also contribute to the high dropout rate from treatment. (iii) Recent studies report that along with effective medical treatment, patients also seek assisted reproductive care with a patient-centered orientation [4, 8, 17].

Considering the above, insights are required into the patient’s perspective on infertility care. Reproductive medicine must focus on other quality dimensions besides ‘effectiveness’ (pregnancy rate), and in particular, on the patient’s satisfaction with the care provided.

Assessing patients’ experiences and needs with regard to fertility services and facilities can provide these insights through the patients’ eyes and help healthcare staff better understand their patients’ needs, preferences, and wishes [18]. They could shed light on the weaknesses and strengths of the care as currently delivered and highlight patients’ needs as well; making this perspective known to healthcare professionals may lead to a noticeable improvement in the quality of care [19–21].

Patients’ experiences are being increasingly regarded as a vital component in improving the delivery of quality healthcare services [22, 23]. Patient satisfaction is typically valuated through interviews [24, 25] and questionnaires [26–31]. This can be particularly important if professionals’ perceptions of their patients’ experiences with care do not accurately reflect the actual state of affairs [32], because this may impede their ability to make changes that are truly beneficial to their patients [33–35].

### The present study

Over the last thirty years, Israel has had a significant impact on promoting and using IVF research. Its national health insurance program, universal and compulsory for all Israeli citizens, covers nearly all fertility treatments. Specifically, this coverage includes all IVF costs for the first and second child for all Israeli women. IVF treatments for the third child onward and even private IVF treatments are partially covered by public funds [36].

The aim of this study was: (i) to identify different aspects of satisfaction with fertility care relevant to patients; (ii) to identify gender differences in evaluation of patients’ satisfaction with fertility services and facilities in the fertility clinics; (iii) to identify predictors of satisfaction, and (iv) to determine with what accuracy fertility specialists can assess their patients’ experiences, as a way to measure patients’ satisfaction and quality of care.

## Methods

All analyses were performed using SPSS (version 19.0 for Windows, SPSS Inc., Chicago, IL, USA).

### Study setting

For this study, data was obtained from IVF patients and healthcare professionals in 8 public IVF units in public hospitals in Israel. The patients in this sampling were infertile couples, 142 women and 62 men, who had undergone or were currently undergoing IVF treatment. Healthcare professionals were enlisted to ask patients for their cooperation. Out of 300 questionnaires distributed, 204 were filled in and returned, i.e., a response rate of 68 %.

The sample of healthcare professionals consisted of gynecologists, and fertility nurses from the same 8 public IVF units. Out of 24 questionnaires distributed, 19 were valid. Respondents were asked to answer all of the questions without exception: questionnaires that were not filled out in their entirety were disqualified, yielding a response rate of 79 %.

All the questionnaires were printed out and delivered manually, for both IVF patients and healthcare professionals.

Ethical approval was obtained in advance from the Ethics Committees (Helsinki committees) in each public hospital.

Written informed consent for participation in the study was obtained from each participant in the IVF patient group and from each participant in the group of healthcare professionals.

### Procedure

The research instruments were questionnaires that were constructed for the study by Spiegel et al. see [37] in a three-stage process: (i) For the initial exploratory stage conducting in-depth interviews with eight fertility experts and 40 IVF patients, 30 women and ten men, to identify which items would be included in the research questionnaires; (ii) Pilot study of 40 IVF patients, five from each hospital; (iii) Main survey: Based on findings of the pilot study, the research questionnaires were revised and modified.

The same version of the research questionnaire was distributed to all the healthcare professionals (see also Aarts et al., [32]). In filling out the questionnaire, the professionals were asked to consider the fertility patients who were treated in their clinic.

This paper focuses on the following issues:

#### Evaluation of treatment

In order to evaluate patient satisfaction with IVF treatments and perceptions of it by professionals, this study was based on the research of Gerteis et al. [38] and on the Picker survey instruments that measure the patient’s experience of care in eight dimensions of patient-centeredness (www.pickerinstitute.org).

The following three major dimensions were tested:

1. Coordination and integration of care: Professionalism of fertility clinic staff; attitude and sensitivity of fertility clinic staff and their relationship with patients; no personnel changes in the fertility clinic staff from beginning of treatment to the end; provision of consulting services and follow-up support – (medical, social and psychological factors).
2. Information: Information on the chances of success (taking home a baby); information on prognosis, different treatment options, clinical aspects, and possible side effects of treatment; information about medical issues during pregnancy (multiple pregnancies, ectopic pregnancies, miscarriages, etc.); information about potential health problems of “test tube babies” - defects, prematurity; information on treatment costs.
3. Access to care and Physical conditions: Geographical accessibility; physical conditions in the operating room - new/old medical equipment; physical conditions in the recovery room (number of beds, personal bedside cabinet, location of bathroom, privacy); physical conditions in the waiting room (new/old furniture, drinks available, reading material, newsletters, atmosphere); waiting times; standby time on the waiting list.

The patients’ Evaluation of treatment questions were presented on a 7-point Likert scale in which 1 represents ‘Completely dissatisfied’; 2 represents ‘Mostly dissatisfied’; 3 represents ‘Somewhat dissatisfied’; 4 represents ‘neither satisfied or dissatisfied’, 5 represents ‘Somewhat satisfied’; 6 represents ‘Mostly satisfied’ and 7 represents ‘Completely satisfied’.

In order to enable a comparison of the three major dimensions of satisfaction – of patients and fertility professionals - two methods were used for processing the data:

1. Principal Component Analysis (PCA)
2. Indices construction

### Principal Component Analysis (PCA)

Principal Component Analysis (PCA) is the most widely used extraction method of component analysis and is most appropriate when the purpose is to reduce the number of items to a smaller number of representative components [39, 40].

The number of components to retain is determined by the criteria, which are that each PC explain at least 5 % (5 %-10 %) of the variance; the cumulative variance is at least 50 % .The literature varies on how much variance should be explained before the number of factors is sufficient. The majority suggest that 75–90 % of the variance should be accounted for [41, 42]. However, some indicate as little as 50 % of the variance explained is acceptable [43], and eigenvalues, which indicate the amount of variance explained by each component [42], are greater than one (Kaiser criterion) [44].

All the items relating to satisfaction with treatment were analyzed using PCA, and the analysis yielded three factors of satisfaction: (i) Human factor: satisfaction with coordination and integration of care; (ii) Information factor: satisfaction with information; (iii) Physical factor: satisfaction with access to care and physical conditions

### Indices construction

Following Van Empel et al. [8], a sum score was calculated adding up the accompanying item scores. The dimension sum scores with diverse maxima were transformed into indices from 1.00 (worst possible) to 10.00 (best possible), using the same formula of Van Empel et al. ([8], p.144): “satisfaction index = 9* [(actual sum score - lowest possible sum score)/ (highest possible sum score - lowest possible sum score)] +1.”

Three satisfaction indices were defined here: (i) Human Satisfaction index (ii) Information Satisfaction index (iii) Physical Satisfaction index.

#### General psychological responses of the respondents

The experience of infertile couples is described in the literature as emotionally taxing [45–47]. The unique psychological factors of in-vitro fertilization (IVF) have been examined and assessed in order to discover whether psychological variables are correlated to patient satisfaction in these factors.

The psychological items were formulated as questions, such as ‘To what degree do you experience the following feelings at these times: guilt, success, etc.?’ Each item was analyzed individually and then graded on a five-point Likert scale in which: 1 represents ‘very slightly or not at all’, 2 ‘a little’, 3 ‘moderately’, 4 ‘a lot’ and 5 ‘extremely’

A Principal Component Analysis (PCA) of the psychological responses was conducted; this analysis yielded three psychological factors (i) Pessimism (ii) Activeness: active involvement in obtaining information and making decisions during treatment, taking initiative, and accepting full responsibility for the stages of treatment and results (iii) Shame.

#### Monetary evaluation of a treatment cycle – what is the maximum amount a respondent is willing to pay for a cycle of IVF treatment

The instrument chosen for economic evaluation of IVF treatment was the willingness to pay (WTP) [48].

The foremost economic theory in decision making by consumers posits that individuals try to maximize the utility of the goods and services they receive (subject to certain constraints). According to Lancaster [49, 50], the utility derived by each consumer from the characteristics of the good is different than the utility derived from the good as a whole.

Ryan [51] applied Lancaster’s utility approach to the field of health economics, using the contingent valuation methodology (CVM), which allows the assessment of a non-market good with a complex utility function. This assessment is made using a technique known as Willingness-to-Pay (WTP), where respondents are asked questions directly in a survey about their “Maximum WTP” – the maximum amount which they would be willing to pay for a service/product or an attribute of a service/product not available in a regular market, or non-priced goods and services. WTP is based on the assumption that “the maximum amount of money an individual is willing to pay for a commodity is an indicator of the value to him/her of that commodity” ([52], p. 182).

The respondents were asked to state ‘what is the amount of money they are willing to pay for one IVF treatment?’

The present study sought to check whether the dimensions of patient satisfaction are correlated with the willingness to pay for IVF treatment.

#### Demographic, socio-economic and health characteristics

Questions about socioeconomic position, number of children not from IVF, number of children from IVF, years of infertility, diagnosed infertility, and number of fertility treatments were derived from the baseline questionnaire.

### Statistical analysis

Using a Pearson correlation, each of the three satisfaction factors were correlated with the demographic, socio-economic, health characteristics, psychological factors, and the WTP variable. P-values < 0.05 were considered statistically significant.

Gender differences in the satisfaction indices were assessed using a t-test. Another comparison was made between patients’ experiences and professionals’ perceptions of these experiences. The mean scores of patients and of professionals were compared using t-tests to detect any statistical differences. The group of professionals was taken as one group rather than broken down into physicians and nurses which would have made the group sizes too small. As for significance, *P* < 0.05 was considered statistically significant.

In order to assess the demographic, socio-economic, health characteristics, and psychological factor influence on the satisfaction indices, an Ordinary least squares (OLS) regression was used. As with OLS regression, F Value is the F-statistic signifying the Mean Square Model divided by the Mean Square Error. The F value should be with a p value (Pr > F) smaller than the standard criterion of 0.05.

R-Square is the proportion of variance in the dependent variable which can be explained by the independent variables. This is an overall measure of the strength of association and does not reflect the extent to which any particular independent variable is associated with the dependent variable.

In the social sciences, low R-squared values are common and expected. “Micro data on individuals, families, or households tend to have low R-squared because there is so much variation in individual behavior. Low R-squared do not necessarily mean that the model is poor” [53]; p 43. For example, Levitt [54] reports R-squared in the range of 0.06 and 0.37. In the present study, the acceptable R-squared were in the range of 0.04 and 0.1.

Adj R-Sq is a modification of the R-squared that penalizes the addition of external predictors to the model. In the social sciences, Adjusted R-squared is also used for a measure of effect size [55]: small effect 0.0196, medium effect 0.1300, and large effect 0.2600. Savage [56] reports adjusted R-squared in the range of 0.05 to 0.1. In the present study, the acceptable adjusted R-squared values were in the range of 0.03 and 0.1.

## Results

The research questionnaires were composed of questions relating to patients’ experiences with fertility care, and patients’ willingness to pay for IVF treatment and its attributes. Answers regarding Demographic, Socio-Economic and Health Characteristics are presented in Table 1.

**Table 1:** Patients -Socio-Demographic and Health Characteristics of the Sample (%) ^a^

Variables	Females	Males
Age		
21–18	-	-
30–22	29.58	16.13
35–31	28.87	40.32
40–36	25.35	32.26
51–41	16.20	11.29
+ 52	-	-
Degree of Religious Observance ^b^		
Religious ^c^ - Variety of Orthodox	20.42	25.81
Secular ^d^ - Not religiously observant	49.30	40.32
Traditional ^f^ - Observant of some of the religious tradition	30.28	33.87
Education		
Elementary School - 1^st^ grade – 9^th^ grade, age range 6–15	2.11	3.23
High School - 10^th^ grade – 12^th^ grade, age range 16–18	32.39	51.61
Academic degree- College, university	65.49	45.16
Occupation		
Academic ^f^ - Professions that require to earn an academic degree	46.48	40.32
Non-professional ^g^ - Unskilled labor	37.32	51.61
Professional non-academic ^h^ - Skilled labor	16.20	8.06
Personal monthly income $		
< $1,902	56.34	41.94
$1,902–$3,533	32.39	37.10
$3,533+	11.27	20.97
Household monthly income $		
< $2,717	39.44	48.39
$2,717–$5,435	42.25	35.48
$5,435+	18.31	16.13
Family Status		
Unmarried	16.20	
Married	83.80	100.0
# of Children Not From IVF		
0	78.87	83.87
1	17.61	8.06
2+	3.52	8.06
# of Children From IVF		
0	71.83	69.35
1	19.72	19.35
2+	8.45	11.29
Years of infertility		
0–1	38.03	32.26
1–2	34.51	41.94
2–3	16.90	11.29
3–4	5.63	6.45
4–5	2.82	6.45
5–10	2.11	1.61
Diagnosed infertility		
Endometriosis	5.63	4.84
Male factor	32.39	48.39
Mechanical reason - tubal factor	17.61	24.19
Unexplained infertility	44.37	22.58
Number of fertility treatments		
1–2	40.85	51.61
3–4	17.61	17.74
5–7	24.65	16.13
8–10	10.56	8.06
11+	6.34	6.45

^a^[37] “With kind permission from Springer Science + Business Media: Journal of Public Health, Economic implications of in vitro fertilization using willingness to pay, 21(6), 2013, 535-557, Uriel Spiegel & Limor Dina Gonen & Joseph Templeman, Appendix 2: Tables 18 and 19, and any original (first) copyright notice displayed with material”

^b^In Israel, religious observance is a demographic factor that is used widely as a way for people to define themselves regarding their beliefs and practices. This is relevant when dealing with matters of reproduction, which are regulated and circumscribed by religious law and doctrine

^c^The term ‘religious’ refers to Jews who follow the traditional Jewish religion

^d^The term “secular” is not strictly defined and it can mean either “not religious” or “convinced atheists”

^e^The term ‘traditional’ covers a wide range of ideologies and levels of observance, and is based on self-definition

^f^Professions that require an academic graduate degree and sometimes additional required professional licensing, registration, and certification to obtain employment (e.g. engineering, law, medicine, nursing, psychology, pharmacy, social work, economics etc.)

^g^Unskilled labor, generally characterized by low education levels. Work that requires no specific education or experience

^h^Skilled labor that does not require an academic degree but usually requires vocational training (e.g. electrician, mechanic, plumber, welder, etc.)

Our study indicates that infertile patients are generally satisfied with the care they receive. The assessment made by fertility specialists of patient satisfaction with fertility services, facilities and quality of care also indicates that they accurately assess their patients as experiencing general satisfaction for these aspects.

For the descriptive statistics of the patients’ and fertility professionals satisfaction dimensions, see Table 2.

**Table 2:** Descriptive Statistics of the Satisfaction Dimensions ^a^ - Patients and Fertility Professionals

	Patients			Fertility Professionals		
	*N* = 204			*N* = 19		
	Mean	Median	Std. Deviation	Mean	Median	Std. Deviation
Professionalism of fertility clinic staff	6.75	7.00	0.57	6.47	7.00	0.69
Attitude and sensitivity of fertility clinic staff and their relationship with patients	6.72	7.00	0.55	6.31	7.00	0.82
No change in the fertility clinic staff from start of treatment to end	6.48	7.00	1.04	5.73	6.00	1.28
Provision of consulting services and follow-up support (medical, social and psychological factors)	5.54	6.00	1.86	5.26	5.00	1.59
Information on the chances of success (taking baby home)	5.63	6.00	1.58	6.00	6.00	0.88
Information on prognosis, different treatment options, clinical aspects and possible side effects of treatment	5.47	6.00	1.61	5.63	6.00	1.21
Information about medical issues during pregnancy (multiple pregnancies, ectopic pregnancies, miscarriages)	5.39	6.00	1.64	5.57	6.00	1.16
Information about potential health problems of “test tube babies” (defects, prematurity)	4.86	5.00	1.83	5.36	6.00	1.21
Information on treatment costs	5.09	6.00	1.95	5.21	6.00	1.81
Geographical accessibility	6.40	7.00	1.17	5.36	6.00	1.83
Physical conditions in the operating room (New/old medical equipment)	5.62	6.00	1.76	4.68	5.00	1.37
Physical conditions in the recovery room (number of beds, personal equipment cabinet, bathroom location, privacy)	5.64	6.00	1.72	5.31	6.00	1.49
Physical conditions in the waiting room (New/Old, drinks available, reading newsletters, atmosphere)	6.07	7.00	1.37	6.31	7.00	1.20
Waiting times	5.55	6.00	1.72	5.94	7.00	1.47
Standby time on the waiting list	5.27	6.00	1.76	5.52	6.00	1.67

^a^ 7-point Likert scale

1 represents ‘Completely dissatisfied’; 2 represents ‘Mostly dissatisfied’; 3 represents ‘Somewhat dissatisfied’; 4 represents ‘neither satisfied or dissatisfied’, 5 represents ‘Somewhat satisfied’; 6 represents ‘Mostly satisfied’ and 7 represents ‘Completely satisfied’

### Evaluation of treatment

The three factors of satisfaction, assessed using Principal Component Analysis (PCA); (i) Human factor (ii) Information factor and (iii) Physical factor, their variances and eigenvalues are presented in Tables 3, 4 and 5 respectively.

**Table 3:** Principal Component Analysis (PCA) - Human factor - satisfaction with coordination and integration of care

Component	Initial Eigenvalues		
	Total	% of Variance	Cumulative %
Professionalism of fertility clinic staff	2.645	66.115	66.115
Attitude and sensitivity of fertility clinic staff and their relationship with patients	0.590	14.758	80.873
No personnel changes in the fertility clinic staff from beginning of treatment to the end	0.487	12.165	93.038
Provision of consulting services and follow-up support (medical, social, and psychological factors)	0.278	6.962	100

**Table 4:** Principal Component Analysis (PCA) - Information factor - satisfaction with information

Component	Initial Eigenvalues		
	Total	% of Variance	Cumulative %
Information on the chances of success (taking home a baby)	3.432	68.644	68.644
Information on prognosis, different treatment options, clinical aspects, and possible side effects of treatment	0.628	12.560	81.204
Information about medical issues during pregnancy (multiple pregnancies, ectopic pregnancies, miscarriages, etc.)	0.434	8.688	89.892
Information about potential health problems of “test tube babies” (defects, prematurity)	0.320	6.409	96.301
Information on treatment costs	0.185	3.699	100

**Table 5:** Principal Component Analysis (PCA) - Physical factor - satisfaction with access to care and physical conditions

Component	Initial Eigenvalues		
	Total	% of Variance	Cumulative %
Geographical accessibility	3.264	54.399	54.399
Physical conditions in the operating room (New/old medical equipment)	0.921	15.356	69.756
Physical conditions in the recovery room (number of beds, personal bedside cabinet, location of bathroom, privacy)	0.913	15.208	84.964
Physical conditions in the waiting room (New/old furniture, drinks available, reading material, newsletters, atmosphere)	0.407	6.777	91.741
Waiting times	0.290	4.831	96.572
Standby time on the waiting list	0.206	3.428	100

The three satisfaction indices, assessed using Indices Construction are (i) Human Satisfaction index (ii) Information Satisfaction index and (iii) Physical Satisfaction index.

The mean and the standard deviation (SD) of the three satisfaction indices respectively for female patients are 9.10 (SD 1.21); 7.29 (SD 2.22); 8.15 (SD 1.72) and for male patients are 8.98 (SD 1.31); 7.77 (SD 1.94); 8.11 (SD 1.75)

T-test analyses showed no significant gender difference with regard to the Human Satisfaction index, the Information Satisfaction index and the Physical Satisfaction index.

The mean and the standard deviation (SD) of the three satisfaction indices respectively for patients are 9.06 (SD 1.24); 7.44 (SD 2.14); 8.14 (SD 1.73) and for professionals are 8.42 (SD 1.25); 7.83 (SD 1.40); 7.78 (SD 1.88).

T-test analyses showed significant difference between patients and professionals with regard to the Human Satisfaction index.

T-test analyses showed no significant difference between patients and professionals with regard to the Information Satisfaction index and the Physical Satisfaction index.

#### General psychological responses of the respondents

The Pearson Correlation between the three psychological factors and the three satisfaction factors yielded the following results:

There is a positive correlation between the Human factor and the psychological factor Activeness (P-value: 0.04).

There is a negative correlation between the Information factor and the psychological factor Pessimism (P-value: 0.00), and there is a positive correlation between the Information factor and the psychological factor Activeness (P-value: 0.05)

There is a negative correlation between the Physical factor and the psychological factor Pessimism (P-value: 0.01)

#### Monetary evaluation of a treatment cycle

The maximum amount that the respondents are willing to pay for a cycle of IVF treatment in Israel was presented in the paper by Spiegel et al. [37].

The average WTP for IVF amongst female IVF patients is $5573.16 (SD $3,664.69) and amongst male IVF patients is $5,694.44 (SD $4,516.09). There is no significant statistical difference between the average WTP for IVF amongst male and female IVF patients (P-value: 0.8400).

The Pearson Correlation between the WTP variable and the three satisfaction factors (assessed using PCA) yielded a negative correlation between the WTP for IVF treatment and the Physical factor – the satisfaction with access to care and physical conditions (P-value: 0.004).

The Pearson Correlation between the WTP variable and the three satisfaction indices (assessed using indices construction), showed a negative correlation between the WTP for IVF treatment and the Physical Satisfaction index (P-value: 0.00).

#### Demographic, socio-economic and health characteristics

The Pearson Correlation analysis indicated that patients with a high personal monthly income were less likely to feel positive satisfaction with the Human factor of coordination and integration of care – (P-value: 0.04).

Patients who had gone through a large number of fertility treatments reported a higher level of satisfaction with the Information factor: information (P-value: 0.001).

Patients with a high level of education, a high personal monthly income, and a high household monthly income were less likely to have positive satisfaction with the Physical factor: access to care and physical conditions – (P-value: 0.006, 0.002, and 0.003 respectively).

T-test analyses showed no significant gender difference with regard to the Human factor (satisfaction with coordination and integration of care), the Information factor (satisfaction with information), and the Physical factor (satisfaction with access to care and physical conditions).

### Regression analysis

The influence of different attributes on each of the satisfaction indices (assessed using indices construction) is commonly estimated by ordinary least squares (OLS) regression techniques.

The dependent variables for the first, second, and third regressions were: Human Satisfaction index; Information Satisfaction index and Physical Satisfaction index, respectively.

The independent variables for the first, second, and third regressions were: degree of religious observance, number of children, education, personal monthly income, household monthly income, number of fertility treatments; psychological factor - Pessimism; psychological factor - Activeness; psychological factor - Shame, gender, and age.

The variables that were found to be significant predictors of the Human Satisfaction index are: age and personal monthly income.

The variables found to be significant predictors of the Information Satisfaction index are: number of fertility treatments, activeness and the psychological factor - pessimism. Satisfaction with information provision was found to be higher among patients who had undergone a higher number of fertility treatments; among patients who were less pessimistic, and among patients who were more active

The variables that were found to be significant predictors of the Physical Satisfaction index are: household monthly income and psychological factor - Pessimism. Physical Satisfaction was found to be higher among patients whose household monthly income was lower, and higher among patients who were less pessimistic.

In Tables 6, 7, and 8 respectively, one can observe the linear models of the Human Satisfaction index, the Information Satisfaction index, and the Physical Satisfaction index.

**Table 6:** Linear Model - Dependent Variable - Human Satisfaction Index

	Patients *N* = 204	
	F Value	4.82
	Pr > F	0.009
	R^2^	0.046
	Adj R^2^	0.036
Variable	Parameter Estimate	Pr > |t|
Intercept	15.98	<.0001
Age	2.21	0.029^*^
Education	0.52	0.60
Personal monthly income	−2.72	0.00*
Household monthly income	0.70	0.48
Degree of religious observance	−0.32	0.75
No. of children	−0.37	0.71
Number of fertility treatments	0.98	0.33
Psychological factor - pessimism	−1.13	0.26
Psychological factor- activeness	1.79	0.08
Psychological factor - shame	−0.48	0.63
Gender	−0.25	0.80

* Significant level ≤0.05

**Table 7:** Linear Model - Dependent Variable - Information Satisfaction Index

	Patients *N* = 204	
	F Value	7.69
	Pr > F	0.000
	R^2^	0.10
	Adj R^2^	0.09
Variable	Parameter Estimate	Pr > |t|
Intercept	31.57	<.0001
Age	−0.67	0.50
Education	−0.90	0.36
Personal monthly income	−1.04	0.30
Household monthly income	−1.35	0.18
Degree of religious observance	0.88	0.38
No. of children	0.17	0.87
Number of fertility treatments	3.33	0.00^*^
Psychological factor - pessimism	−2.60	0.01*
Psychological factor - activeness	2.17	0.03*
Psychological factor shame	0.07	0.94
Gender	1.42	0.16

* Significant level ≤0.05

**Table 8:** Linear Model - Dependent Variable - Physical Satisfaction Index

	Patients *N* = 204	
	F Value	7.86
	Pr > F	0.001
	R^2^	0.07
	Adj R^2^	0.06
Variable	Parameter Estimate	Pr > |t|
Intercept	30.38	<.0001
Age	−0.30	0.76
Education	−1.52	0.13
Personal monthly income	−1.39	0.17
Household monthly income	−3.17	0.00^*^
Degree of religious observance	−1.25	0.21
No. of children	−0.22	0.82
Number of fertility treatments	1.04	0.30
Psychological factor - pessimism	−2.30	0.02*
Psychological factor - activeness	1.25	0.21
Psychological factor - shame	−0.76	0.45
Gender	−0.75	0.45

* Significant level ≤0.05

## Discussion

One of the most important findings to emerge from this study was the high level of satisfaction among IVF patients with the medical care they received. While previous studies have also found high patient satisfaction [30], the findings of the present study go beyond past studies in analyzing the experiences and perceptions of both patients and professionals in public IVF clinics in Israel. Because the State of Israel funds IVF treatments for married couples and for single women until the birth of a second child, it is a useful setting for this type of research. The IVF treatments are given in public clinics, where the physical conditions are the conditions of public hospitals. Because the IVF treatment is publicly rather than privately funded, the IVF units are very busy and pressured. Consequently, personal attention and the detailed information relevant to a specific individual cannot always be given as desired. Hence, the information provided is general and basic, usually touching only upon the medical technical procedure used by the doctors and nurses. This may also apply to the attitude and sensitivity of fertility clinic staff and their relationship toward patients, and their limited ability to provide consultation and support post-treatment. Despite all these constraints, the results of the present study indicate that when it comes to satisfaction with IVF-treatment, the patients are, on the whole, satisfied.

A second important finding was that psychological factors were significantly correlated with patient satisfaction. Improving patient satisfaction with IVF services, treatments, and facilities may have a positive influence on the psychological and mental state of patients (e.g., active involvement in obtaining information and decision making, taking initiative and accepting responsibility for treatment and results) and in turn, affect the outcome of fertility treatments and treatment dropout rates.

Additional findings of interest emerged in relation to the demographic characteristics.

In contrast with the contradictory findings of previous studies regarding the direction of the relationship between age and the patients’ perception of care [28, 57], the findings of this study show age to be a significant predictor of satisfaction with the coordination and integration of care. Contrary to previous findings e.g., [58], patients who already had children were not more likely than patients without children to have a positive perspective on the medical care they received. The findings relating to medical characteristics, too, differed from the conclusions of other studies [29, 59] which had found that length of infertility was positively related to the patients’ perspective on care. The present study indicates that there was no statistically significant correlation between duration of infertility and satisfaction with care. The correlation between source of infertility (male infertility–female infertility) and the patients’ perspective on care showed no significant results in previous studies i.e. [30, 60]. In the current study, however, patients with a high education level and high income were less likely to have a positive perspective on IVF care in terms of satisfaction. For example, they were less likely to be satisfied with the physical environment. A possible explanation may be based on the understanding that satisfaction is a customer’s post-purchase evaluation of a product/service offering [61]. Customer satisfaction is evaluated by comparing expectation with performance: when performance exceeds expectation, the customer is satisfied. Dissatisfaction is the result of expectations being higher than actual performance [62]. The customer’s evaluation of the service received is influenced by the quality of service as he perceived it ([63] Grönroos). The health care environment has changed greatly in the last decades; these changes range from modified consumer needs and wants to an increased number of payouts by health insurance companies and other third parties. Consequently, the private hospitals have used the opportunity to gain a competitive advantage by, among others, improving the non-medical physical environment. Thus, people with higher levels of education and income who used private hospitals (because they could effort to pay for treatments privately or for private insurance) perceive the physical environment in private hospitals as being superior to that of public hospitals.

As Schmidt et al. [30] found, patients of lower socio-economic status here, too, reported more satisfaction with both medical and patient-centered treatment than patients from higher socio-economic levels. This finding may be interpreted as an indication that the fertility clinic staff was able to meet the needs of patients with relatively fewer social resources. Another possible interpretation may be that people from lower social classes tend to view medical staff less critically and more reverentially, hence, they may tend less to offer negative evaluations.

The current study shows a correspondence between healthcare professionals’ perceptions of their patients’ experiences and the patients’ actual experiences as regards information satisfaction and satisfaction with the physical conditions.

Regarding satisfaction with coordination and integration of care, professionalism of fertility clinic staff, attitude and sensitivity of fertility clinic staff and their relationship with patients, provision of consulting services and follow-up support (medical, social, and psychological factors), this study shows that the healthcare professionals’ perceptions of their patients’ experiences with fertility care do not correspond with the patients’ actual experiences. Healthcare professionals underrated their own performance. A possible explanation could be that healthcare professionals think that patients consider their performance mainly in relation to and in light of the percentage of successes. Since the percentage of successes in IVF treatment is relatively low (about 20 %-25 %^1^), healthcare professionals tend to give lower ratings to their own performance than do their patients.

In considering the findings of this study, some limitations must be addressed.

First, the selection bias caused by the under-representation of patients from public clinics where the treatment is paid by public funding rather than patients themselves. Patients who are granted free treatment may be less critical.

Second, the sampling of professionals was not random, since there is a limited number of physicians and nurses working in IVF clinics. The sample who participated in this study might not be fully representative of the total group of fertility professionals in Israel. Nonetheless, the high response rate (79 %) may compensate for this selection bias.

## Conclusions

In summary, patients’ satisfaction with medical care is increasingly acknowledged to be one of the fundamental dimensions of quality of care, and particularly so when it comes to treatment in aid of infertility [4, 17]. Patient satisfaction should be taken into account in evaluating fertility treatments and other medical interventions.

By assessing patients’ experiences and needs, insights into the quality of care through the patients’ eyes can be gained which may help healthcare staff understand their patients’ preferences, wishes, and needs. Acknowledging the importance of patient satisfaction may shift power towards patients and therefore requires a change in the mindset of professionals [19, 64–66].

### Ethics, consent and permissions

Ethical approval was obtained in advance from the Ethics Committees (Helsinki committees) in each public hospital.

Written informed consent for participation in the study was obtained from each participant in the IVF patient group and from each participant in the group of healthcare professionals.

### Consent to publish

I have obtained from the participant consent to publish to report individual patient data.

## Footnotes

1 Ministry of Health –State of Israel
http://www.health.gov.il/PublicationsFiles/English/IVF-handbook_EN.pdf.
